# Integrated Characterization of MicroRNA and mRNA Transcriptome in Papillary Thyroid Carcinoma

**DOI:** 10.3389/fendo.2018.00158

**Published:** 2018-04-16

**Authors:** Azliana Mohamad Yusof, Rahman Jamal, Rohaizak Muhammad, Shahrun Niza Abdullah Suhaimi, Isa Mohamed Rose, Sazuita Saidin, Nurul-Syakima Ab Mutalib

**Affiliations:** ^1^UKM Medical Molecular Biology Institute (UMBI), Universiti Kebangsaan Malaysia, Kuala Lumpur, Malaysia; ^2^Department of Surgery, Faculty of Medicine, Universiti Kebangsaan Malaysia, Kuala Lumpur, Malaysia; ^3^Department of Pathology, Faculty of Medicine, Universiti Kebangsaan Malaysia, Kuala Lumpur, Malaysia

**Keywords:** microRNA, papillary thyroid carcinoma, BRAF V600E, transcriptome sequencing, integrated analyses

## Abstract

The incidence rate of papillary thyroid carcinoma (PTC) has rapidly increased in the recent decades, and the microRNA (miRNA) is one of the potential biomarkers in this cancer. Despite good prognosis, certain features such as lymph node metastasis (LNM) and *BRAF* V600E mutation are associated with a poor outcome. More than 50% of PTC patients present with LNM and *BRAF* V600E is the most common mutation identified in this cancer. The molecular mechanisms underlying these features are yet to be elucidated. This study aims to elucidate miRNA–genes interaction networks in PTC with or without LNM and to determine the association of *BRAF* V600E mutation with miRNAs and genes expression profiles. Next generation sequencing was performed to characterize miRNA and gene expression profiles in 20 fresh frozen tumor and the normal adjacent tissues of PTC with LNM positive (PTC LNM-P) and PTC without LNM (PTC LNN). *BRAF* V600E was genotyped using Sanger sequencing. Bioinformatics integration and pathway analysis were performed to determine the regulatory networks involved. Based on network analysis, we then investigated the association between miRNA and gene biomarkers, and pathway enrichment analysis was performed to study the role of candidate biomarkers. We identified 138 and 43 significantly deregulated miRNAs (adjusted *p* value < 0.05; log2 fold change ≤ −1.0 or ≥1.0) in PTC LNM-P and PTC LNN compared to adjacent normal tissues, respectively. Ninety-six miRNAs had significant expression ratios of 3p-to-5p in PTC LNM-P as compared to PTC LNN. In addition, ribosomal RNA-reduced RNA sequencing analysis revealed 699 significantly deregulated genes in PTC LNM-P versus normal adjacent tissues, 1,362 genes in PTC LNN versus normal adjacent tissue, and 1,576 genes in PTC LNM-P versus PTC LNN. We provide the evidence of miRNA and gene interactions, which are involved in LNM of papillary thyroid cancer. These findings may lead to better understanding of carcinogenesis and metastasis processes. This study also complements the existing knowledge about deregulated miRNAs in papillary thyroid carcinoma development.

## Introduction

Papillary thyroid cancer (PTC) is the most common type of thyroid malignancy and it accounts for 80% of thyroid cancers; with approximately 77% diagnosed in women (1). Women are three to four times more likely to develop thyroid cancer than men, an opposite trend compared to other non-gender-specific sites (2). In the past few decades, its occurrence has increased significantly in many countries (3). Despite a high survival rate, patients with certain clinicopathologic features have been associated with poorer prognosis, such as older age at diagnosis (4), gender (5), extrathyroidal invasion (6), *BRAF* V600E mutation status (7), and lymph node metastasis (LNM) (8). In addition, the presence of LNM is associated with locoregional recurrence and increases the risk of mortality even in young patients (8). LNM is one of the factor of PTC propensity to metastasize to distant organs (9). Due to that, lymph node resection in PTC patients is considered vital (10).

The molecular mechanisms underlying LNM in PTC remain poorly understood. One of the molecules of interest in terms of elucidating the molecular landscape of cancer is microRNA (miRNA). miRNAs are small non-coding RNAs that comprise of 19 to 22 nucleotides, which negatively regulate gene expression. The relationship between miRNA with tumor growth, disease progression, and response to treatment has been demonstrated through miRNA expression profiling studies, which indicate the ability of these molecules to be used as biomarkers for cancer diagnosis and prognosis (11). miRNA expression is not only able to distinguish between cancer samples and normal samples from various types of cancer, but it can also distinguish whether the cancer cases have metastasized to the lymph nodes or not (12). In addition, the involvement of miRNA in the process of tumorigenesis and metastasis in PTC has also been demonstrated (13). Furthermore, the integration of miRNA and mRNA analysis can assist in detecting potential targets and may show the evidence to explain the importance of biological association of LNM in PTC (14).

The knowledge of how miRNAs interact with their target mRNAs and the consequences of this interaction will bring us one step closer to understanding the biological impact of these regulation on cell fate and function. Therefore, this study aims to elucidate miRNA–genes interaction networks in PTC with and without LNM and to associate *BRAF* V600E mutation with miRNAs and genes expression profiles.

## Materials and Methods

### Clinical Specimens

Ten pairs of tumor and adjacent normal fresh frozen tissues were collected from patients diagnosed with PTC with or without LNM (PTC LNM-P and PTC LNN, respectively) from the Universiti Kebangsaan Malaysia Medical Centre (UKMMC). This study was carried out in accordance with the recommendations of UKM Research Ethics Committee (UKMREC; UKM 1.5.3.5/244/UMBI-2015-002). Written informed consent was obtained from all the study participants. The tissues were dissected, snap-frozen, and stored in liquid nitrogen. All tissues were cryosectioned and stained with hematoxylin and eosin and the percentage of tumor and normal cells were assessed by a pathologist (Isa Mohamed Rose). Only cancer samples with at least 80% cancerous cells and normal adjacent thyroid tissues with less than 20% necrosis were selected for further analysis. The 10 paired fresh frozen specimens were subjected to miRNA and mRNA sequencing while additional 11 formalin-fixed, embedded tissues (FFPE) samples were used for qPCR validation.

### Nucleic Acid Isolation

All fresh frozen tissues and FFPE specimens were subjected to nucleic acid extraction using Allprep DNA/RNA/miRNA Universal Kit or Allprep DNA/RNA FFPE Kit (Qiagen, Germany), respectively according to manufacturer’s recommendations. The integrity of RNA was assessed using Agilent RNA 6000 Nano Kit and only total RNA with RNA Integrity Number (RIN) of at least six were used for subsequent steps. The miRNA content was quantified using Small RNA Kit (both from Agilent Technologies, Palo Alto, CA, USA) while the quality of DNA was assessed using 1% agarose gel. The quantity and purity of RNA and DNA were assessed using Qubit 2.0 fluorometer (Invitrogen, USA) and Nanodrop 2000c Spectrometer (Thermo Scientific/Nanodrop, DE), respectively. The acceptable purity as indicated by A260/280 is 2.0–2.2 for the RNA and 1.8–2.0 for the DNA.

### Library Preparation

microRNA libraries were prepared using the TruSeq Small RNA Sample Prep Kit (Illumina, San Diego, CA, USA). Briefly, 3′ and 5′ adapters were sequentially ligated to the ends of small RNAs fractionated from 2 µg of total RNA, and reverse transcribed to generate cDNA. The cDNA was amplified using a common primer complementary to the 3′ adapter, and a primer containing 1 of 48 index sequences. Samples were size-selected (140–160 bp fragments) on a 6% polyacrylamide gel, purified, quantified, normalized to 2 Nm, and pooled for multiplexed sequencing.

Meanwhile, whole transcriptome libraries were prepared using Ion Total RNAseq v2 kit (Life Technologies, USA) according to manufacturer’s protocol. Two micrograms of total RNA were used for ribosomal removal using RiboMinus Eukaryote kit (Life Technologies, USA). ERCC RNA Spike-In Control Mixes (Thermo Scientific, USA) were added into rRNA depleted-RNA before RNA fragmentation using RNase III. rRNA-depleted and fragmented RNA were quantified using Qubit fluorometer assay and Agilent RNA 6000 Nano Kit. Subsequently, the fragmented RNA was hybridized and ligated to adaptors, reverse-transcribed, purified, size-selected, and amplified. The final libraries were quantified using Agilent High Sensitivity DNA chip (Agilent Technologies, USA) and were normalized to 100–130 pM for the next step.

### miRNA and mRNA Sequencing

For miRNA sequencing, the resulting pooled libraries were hybridized to oligonucleotide-coated single-read flow cells for cluster generation using HiSeq^®^ Rapid SR Cluster Kit v2 on Hiseq 2500 (Illumina, USA). The clustered pooled miRNA libraries were then sequenced on the HiSeq 2500 for 50 sequencing cycles using HiSeq^®^ Rapid SBS Kit v2 (50 Cycle) (Illumina, USA). As for mRNA sequencing, each library were subjected to clonal amplification on Ion Chef System using Ion PI IC 200 kit followed by sequencing using PI BC v2 chip on Ion Proton system (all from Life Technologies, USA). Two to three mRNA libraries were pooled into a single chip.

### Bioinformatics, Integrative, and Statistical Analyses

Preprocessing of miRNA sequencing data was executed in BaseSpace software (Illumina, USA), and FASTQ files were generated. MiRNA Analysis app version 1.0.0 was used for determination of differentially expressed (DE) miRNAs using the workflow described by Cordero et al. (15). Briefly, the pipeline includes 3′ end adapter removal using cutadapt (16), annotation to miRBase v21 (17), mapping using SHRIMP aligner (18), and differential analysis of miRNAs using DESeq2 (19). In addition, Rank Product statistics were used to detect 3p/5p ratio changes on the same miRNA in two different conditions (20). Unsupervised hierarchical clustering and heatmaps were performed and created using Morpheus from Broad Institute (https://software.broadinstitute.org/morpheus/).Pathway enrichment analysis were executed using DIANA-miRPath v3.0 (21) and significance was determined using Fisher’s Exact Test.

For mRNA sequencing, preprocessing of data were conducted on Torrent Server using Torrent Suite v4.4.2 software (Life Technologies, USA) and FASTQ files were generated and then exported for data analysis using CLCBio Genomics Workbench v8.3 (CLC Bio, Denmark). The manufacturer’s analysis pipeline for transcriptome sequencing was used for differential expression of mRNA. Briefly, the pipeline includes removal of 3′ adapter, mapping to reference transcript (hg19) and differential expression analysis using the Empirical analysis of DGE (EDGE) (22). Only DE miRNAs or genes with *p*-value <0.05 and log2-fold change ≤−1.0 or ≥1.0 were subjected to further analysis.

Integrated miRNA and mRNA analysis were performed using MAGIA2 (23). Log2 normalized reads from the significant DE miRNAs and mRNAs were uploaded into MAGIA2 and positive and negative correlation analyses between miRNAs, transcription factors, and target mRNA were performed. Skip variability filter option was selected because the data uploaded only contain the significant miRNAs or mRNAs. miRNA targets were predicted using DIANA microT (24) (score threshold at 2.0, top 75% of the predictions distribution) and Spearman correlation coefficient was used as correlations measure (threshold <0.05). In addition, prediction of miRNA-transcription factor was also performed based on mirGen v2.0 (25) and TransmiR (26) databases.

### *BRAF* V600E Genotyping

PCR amplification of genomic regions of interest was performed using *BRAF* V600E forward primer 5′-TGCTTGCTCTGATAGGAAAATG-3′ and reverse primer 5′-AGCATCTCAGGGCCAAAAAT-3′ (27). Amplification was performed in a reaction volume of 25 μl containing 50 ng DNA template, 10 μM each primer (Integrated DNA Technologies, USA), 10× PCR Gold Buffer without MgCl_2_, dNTP Mix (10 mM), MgCl_2_ solution (25 mM), AmpliTaq Gold^®^ (5 U/μl) (Applied Biosystems, USA) and nuclease-free water. PCR conditions were as follows; 95°C for 4 min; 35 cycles of 95°C for 45 s, 50°C for 30 s, and 72°C for 1 min; 72°C for 5 min; and held at 4°C. PCR products were visualized by electrophoresis on 1.5% agarose gel with an expected size of ~228 bp. PCR purification was conducted using QIAquick PCR Purification Kit (Qiagen, Germany) as per manufacturer’s instruction. Subsequently, DNA sequencing was performed using ABI Prism 3130xl Genetic Analyzer (Applied Biosystem, USA).

### Real-Time PCR Validation

Based on 3p/5p miRNA expression ratio analysis, six most significant DE miRNAs were chosen for validation. The Pick-&-Mix panel (Exiqon, Denmark) consisting of hsa-miR-193a-3p, hsa-miR-193a-5p, hsa-miR-376a-3p, hsa-miR-376a-5p, hsa-miR-205-3p, and hsa-miR-205-5p primers was used on the set of samples with hsa-miR-10b-5p and hsa-miR-191-5p as reference miRNA. This assay was carried out on CFX96 Touch Real-Time PCR Detection (BioRad, USA). Fold change was calculated using 2^−ΔΔCq^ (28) and statistical correlation between miRNA sequencing and qPCR was analyzed using *R*^2^ on GraphPad Prism v7.0 (Graphpad Software, USA).

## Results

### Demographic Data

As summarized in Table 1, all subjects in our discovery phase were female patients and 70% of them were Malays. Mean age at diagnosis was 51.6 years in the PTC LNM-P group and 47.4 years in the PTC LNN group. Majority of the cancers were located at the right lobe. All subjects with PTC LNM-P had *BRAF* V600E mutation.

**Table 1:** Clinicopathological features of patients in discovery and validation phase.

Clinicopathological features	Discovery phase		Validation phase	
	Papillary thyroid carcinoma (PTC) LNM-P *n*(%)	PTC LNN *n*(%)	PTC LNM-P *n*(%)	PTC LNN *n*(%)
**Gender**				
Male	0 (0)	0 (0)	4 (57.1)	2 (50)
Female	5 (100)	5 (100)	3 (42.9)	2 (50)
**Ethnic**				
Malay	3 (60)	4 (80)	6 (85.7)	2 (50)
Chinese	2 (40)	1 (20)	1 (14.3)	1 (25)
Indian	0 (0)	0 (0)	0 (0)	1 (25)
**Age at diagnosis, year**				
≥45 years old	4 (80)	4 (80)	5 (71.4)	2 (50)
<45 years old	1 (20)	1 (20)	2 (28.6)	2 (50)
Mean	51.6	47.4	43.4	41.5
**Tumor location**				
Right lobule	3 (60)	3 (60)	4 (57.1)	1 (25)
Left lobule	1 (20)	1 (20)	3 (42.9)	3 (75)
Isthmus	1 (20)	1 (20)	0 (0)	0 (0)
**Tumor size (cm)**				
≤1	0 (0)	2 (40)	0 (0)	1 (25)
>1	5 (100)	3 (60)	7 (100)	3 (75)
***BRAF* V600E mutation**				
Positive	5 (100)	0 (0)	6 (85.7)	2 (50)
Negative	0 (0)	5 (100)	1 (14.3)	2 (50)

### DE miRNAs

**Figure 1:**
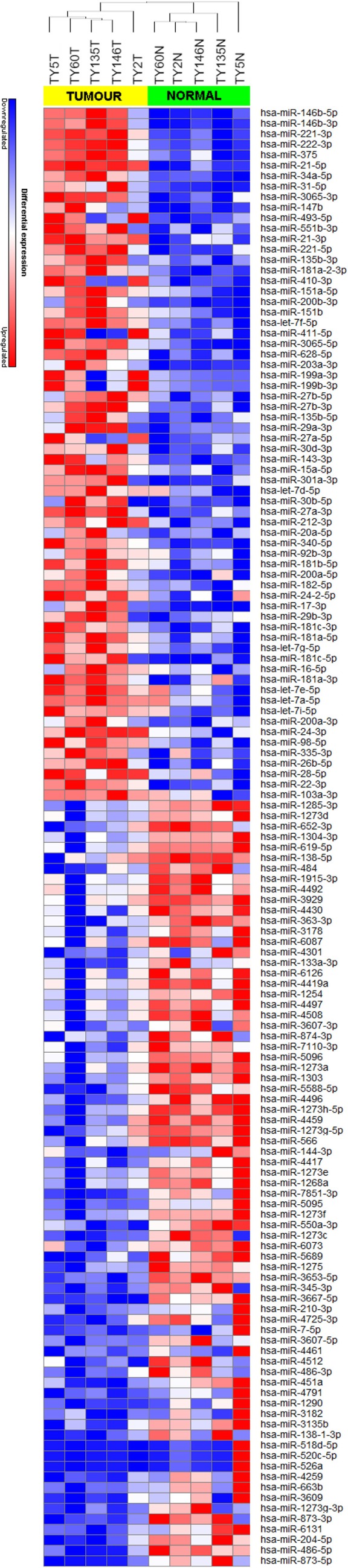
Hierarchical clustering and heatmap for microRNA (miRNA) expression profile in papillary thyroid carcinoma LNM-P versus adjacent normal comparison group. The list of miRNA were filtered with *p*-value <0.05 and log2-fold change ≤−1.0 or ≥1.0. A total of 138 miRNA have significant differentially expression. The sample labeled in yellow bar represents tumor sample while green bar represents normal samples. Color key illustrate the relative expression of miRNA in all samples. Red indicates high expression level while blue indicate low expression level. Dendrogram shows all samples were clustered well according to their respective groups.

**Figure 2:**
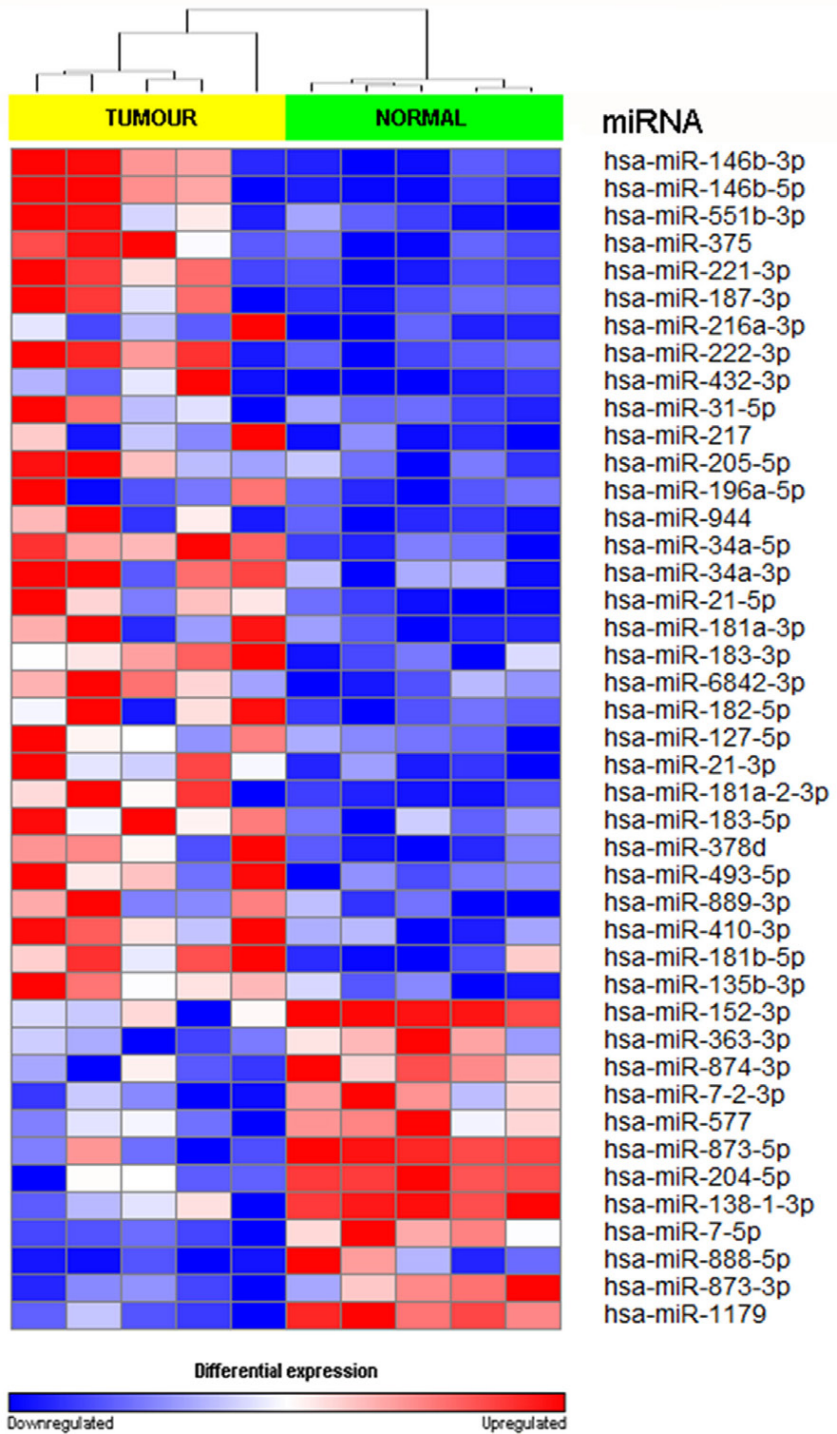
Hierarchical clustering and heatmap for microRNA (miRNA) expression profile in papillary thyroid carcinoma LNN versus adjacent normal comparison group. The list of miRNA were filtered with *p* value <0.05 and log2-fold change ≤−1.0 or ≥1.0. A total of 43 miRNA have significant differentially expression. The sample labeled in yellow bar represents tumor sample while green bar represents normal samples. Color key illustrate the relative expression of miRNA in all samples. Red indicates high expression level while blue indicate low expression level. Dendrogram showed that all samples were clustered well according to their respective groups.

We achieved an average of 5.6 M reads per sample with Q30 (3,779, 968–8,308, 285 reads). miRNA expression analysis was divided into three comparisons, which were PTC LNM-P versus adjacent normal, PTC LNN versus adjacent normal, and PTC LNM-P versus PTC LNN. A total of 138 miRNAs were significantly DE (66 upregulated and 72 downregulated) in PTC LNM-P versus their normal adjacent group. Whereas for PTC LNN versus adjacent normal, 43 miRNAs were significantly DE (31 upregulated and 12 downregulated). However, no miRNA was DE when we compared PTC LNM-P versus PTC LNN. Unsupervised hierarchical clustering revealed that all samples were clustered according to their respective groups (Figures 1 and 2), proving that the miRNAs expression signatures were able to differentiate tumors from normal thyroid tissues even when the tissues originated from the same patients. The list of significant DE miRNAs was provided in Tables S1 and S2 in Supplementary Material. The miRNA sequencing data in fastq. format was deposited at the NCBI Sequence Read Archive (SRA) at http://www.ncbi.nlm.nih.gov/sra with submission IDs SUB2458655 and SUB2753230.

### Pathway Enrichment Analysis of the Deregulated miRNAs

**Figure 3:**
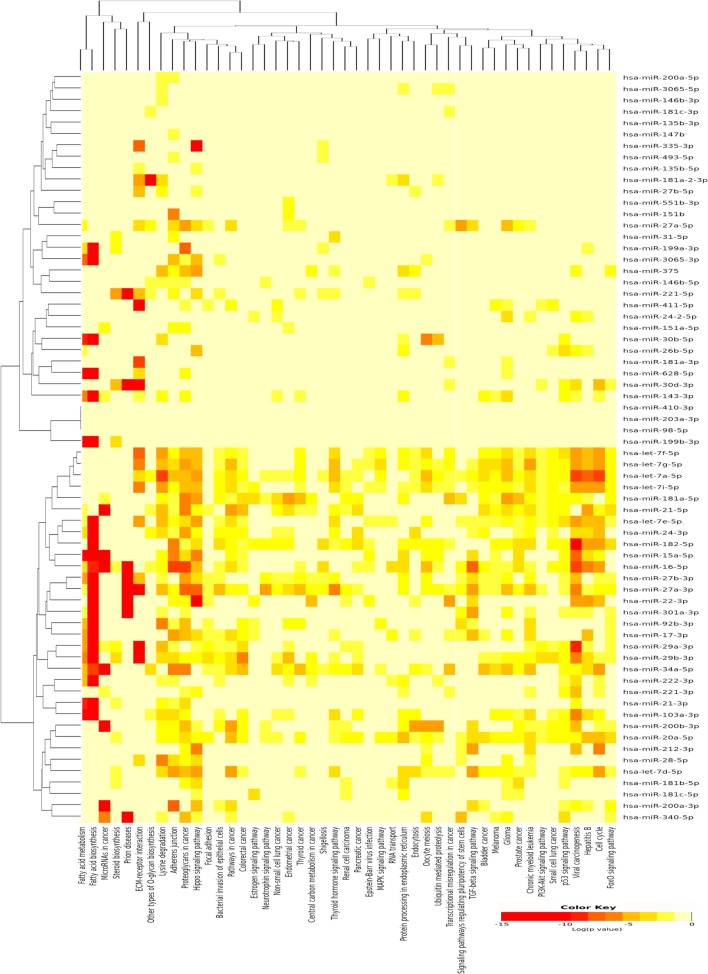
Pathway enrichment analysis on 66 upregulated microRNA (miRNA) in papillary thyroid carcinoma LNM-P and heatmap for miRNA versus pathway (clustered based on their significance value) using DIANA miRPath v3.0 software. Dendrogram on both axis represent hierarchical clustering resulting from miRNA and pathway. The miRNA were clustered together with similar pathway pattern and the pathways were clustered together based on related miRNAs.

A single miRNA may play a specific role in the pathogenesis of PTC; however, the miRNA pathway as a whole may also be of importance. Therefore, the DIANA-mirPath v3.0 (21) was used to identify pathways that are potentially regulated by the expression of multiple miRNAs and to incorporate miRNAs into molecular pathways using experimentally validated miRNA target according to TarBase issue v7 (29). Using the upregulated miRNAs in PTC LNM-P, 47 pathways were enriched significantly (Figure 3). On the other hand, using downregulated miRNAs in PTC LNM-P, four pathways were enriched significantly, which include fatty acid biosynthesis (*p* < 1E−325), fatty acid metabolism (*p* < 1E−325), fatty acid elongation (*p* = 9.30E−10), and fatty acid degradation (*p* = 3.60E−02).

**Figure 4:**
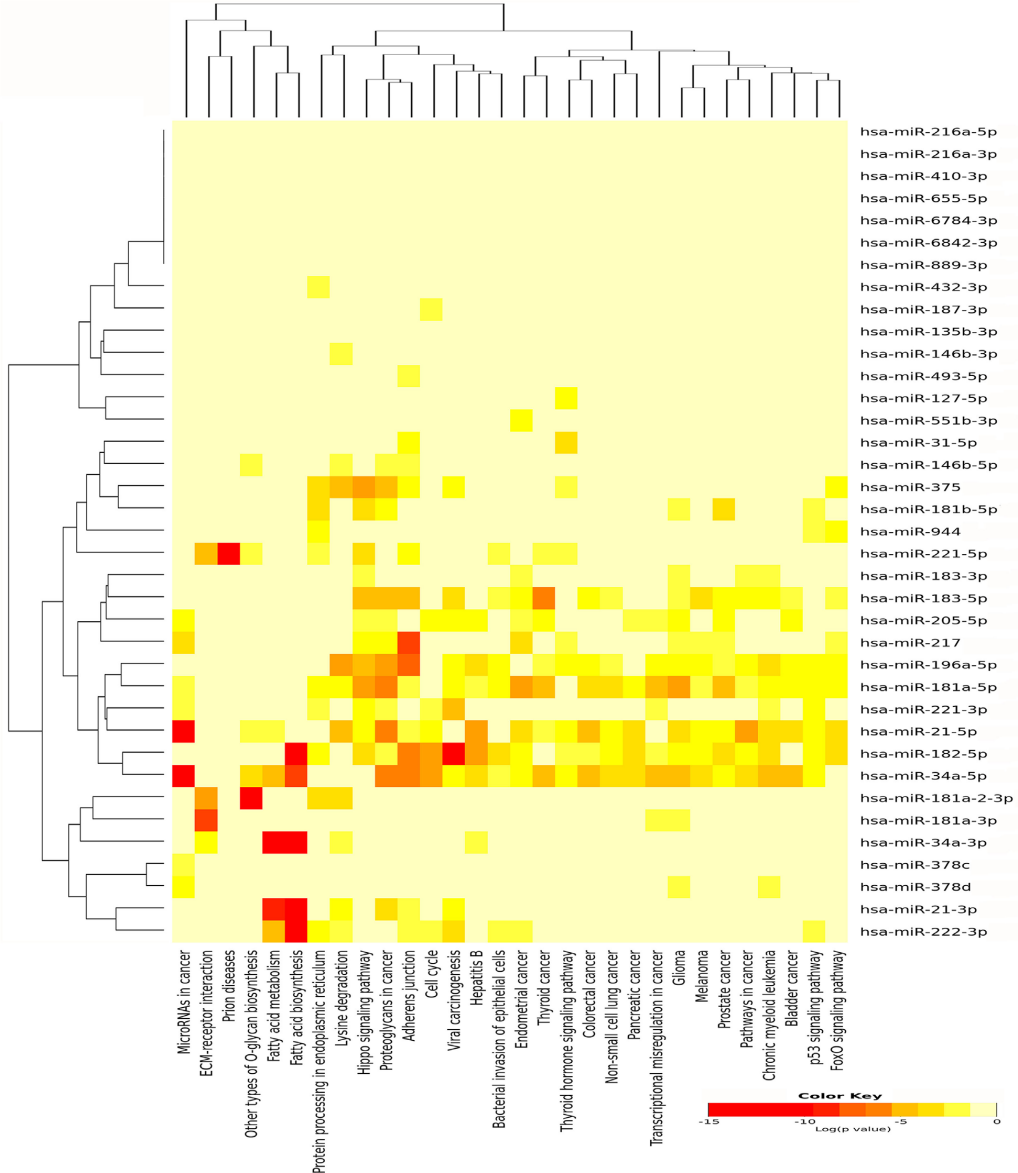
Pathway enrichment analysis on 31 upregulated microRNA (miRNA) in papillary thyroid carcinoma LNN and heatmap for miRNA versus pathway (clustered based on their significance value) using DIANA miRPath v3.0 software. Dendrogram on both axis represent hierarchical clustering resulting from miRNA and pathway. The miRNA were clustered together with similar pathway pattern and the pathways were clustered together based on related miRNAs.

For miRNAs, which were upregulated in PTC LNN, 30 pathways were significantly enriched (Figure 4) while eight pathways were enriched significantly using downregulated miRNAs, which include fatty acid biosynthesis (*p* < 1E−325), fatty acid metabolism (*p* = 1.31E−11), lysine degradation (*p* = 1.35E−11), proteoglycans in cancer (*p* = 9.75E−04), chronic myeloid leukemia (*p* = 3.55E−03), viral carcinogenesis (*p* = 8.51E−03), glioma (*p* = 2.13E−02), and pathways in cancer (*p* = 2.48E−02).

### Ratio of 3p/5p miRNA

**Figure 5:**
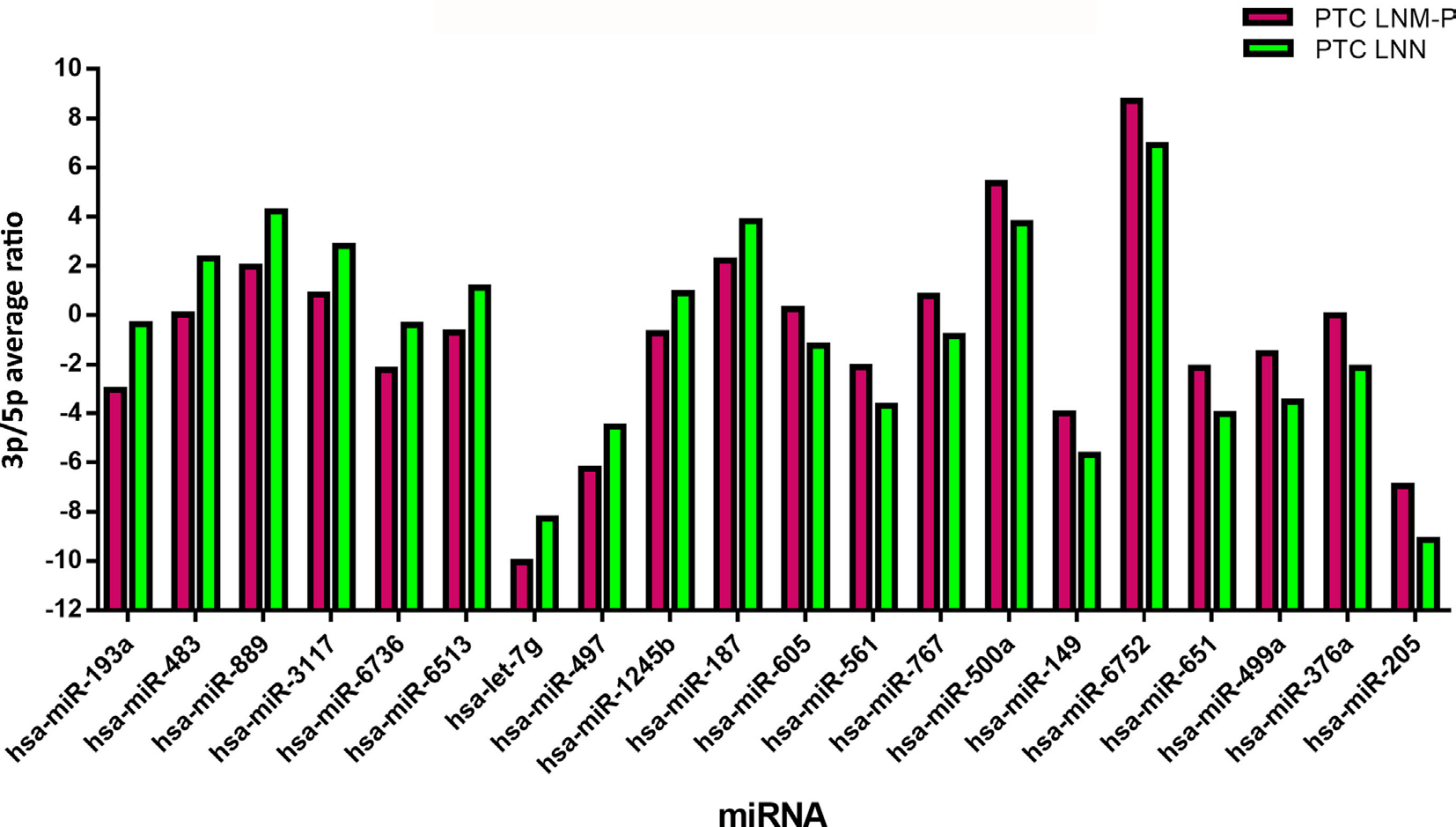
The top 20 3p/5p expression ratio in both comparison groups. Several microRNA (miRNA) have significant expression ratio shows that arm selection preference in miRNA are different significantly between papillary thyroid carcinoma (PTC) LNM-P and PTC LNN.

In order to analyze the 3p and 5p expression ratio on the same miRNA in two different groups, PTC LNM-P and PTC LNN tumor sequencing data were analyzed using RankProd (20). Ninety-six miRNA had significant differential expression ratio and present on both group. Table 2 and Figure 5 show the list of top 20 miRNAs with significant 3p/5p ratio.

**Table 2:** The list of top rank 20 microRNA (miRNA) with highest expression ratio.

miRNA	Log_2_ average ratio	3p/5p (papillary thyroid carcinoma (PTC) LNM-P)	Log_2_ average ratio	3p/5p (PTC LNN)	Fold change
hsa-miR-205	−6.95	−9.14	2.18
hsa-miR-376a	0.01	−2.12	2.13
hsa-miR-499a	−1.54	−3.54	1.99
hsa-miR-651	−2.14	−4.03	1.88
hsa-miR-6752	8.72	6.92	1.80
hsa-miR-149	−4.01	−5.70	1.69
hsa-miR-500a	5.37	3.73	1.64
hsa-miR-767	0.80	−0.84	1.64
hsa-miR-561	−2.11	−3.70	1.59
hsa-miR-605	0.24	−1.24	1.49
hsa-miR-187	2.23	3.81	−1.58
hsa-miR-1245b	−0.74	0.90	−1.64
hsa-miR-497	−6.24	−4.53	−1.71
hsa-let-7g	−10.05	−8.29	−1.75
hsa-miR-6513	−0.71	1.12	−1.83
hsa-miR-6736	−2.23	−0.40	−1.84
hsa-miR-3117	0.83	2.82	−1.99
hsa-miR-889	1.97	4.21	−2.24
hsa-miR-483	0.02	2.29	−2.27
hsa-miR-193a	−3.06	−0.36	−2.70

### DE Genes

Analysis of gene expression was also divided into three groups, which are PTC LNM-P versus adjacent normal, PTC LNN versus adjacent normal, and PTC LNM-P versus PTC LNN. In PTC LNM-P versus adjacent normal, 348 genes were upregulated and 351 were downregulated. As for PTC LNN versus adjacent normal group, 396 genes were upregulated and 966 were downregulated. For PTC LNM-P versus PTC LNN, 733 genes were upregulated and 843 were downregulated. Tables S3–S5 in Supplementary Material summarized the list of significant DE genes. The mRNA sequencing data in fastq. format was deposited at the NCBI SRA at http://www.ncbi.nlm.nih.gov/sra with submission ID SUB2756288.

### Integrated Analysis of miRNA and Gene Expression

**Figure 6:**
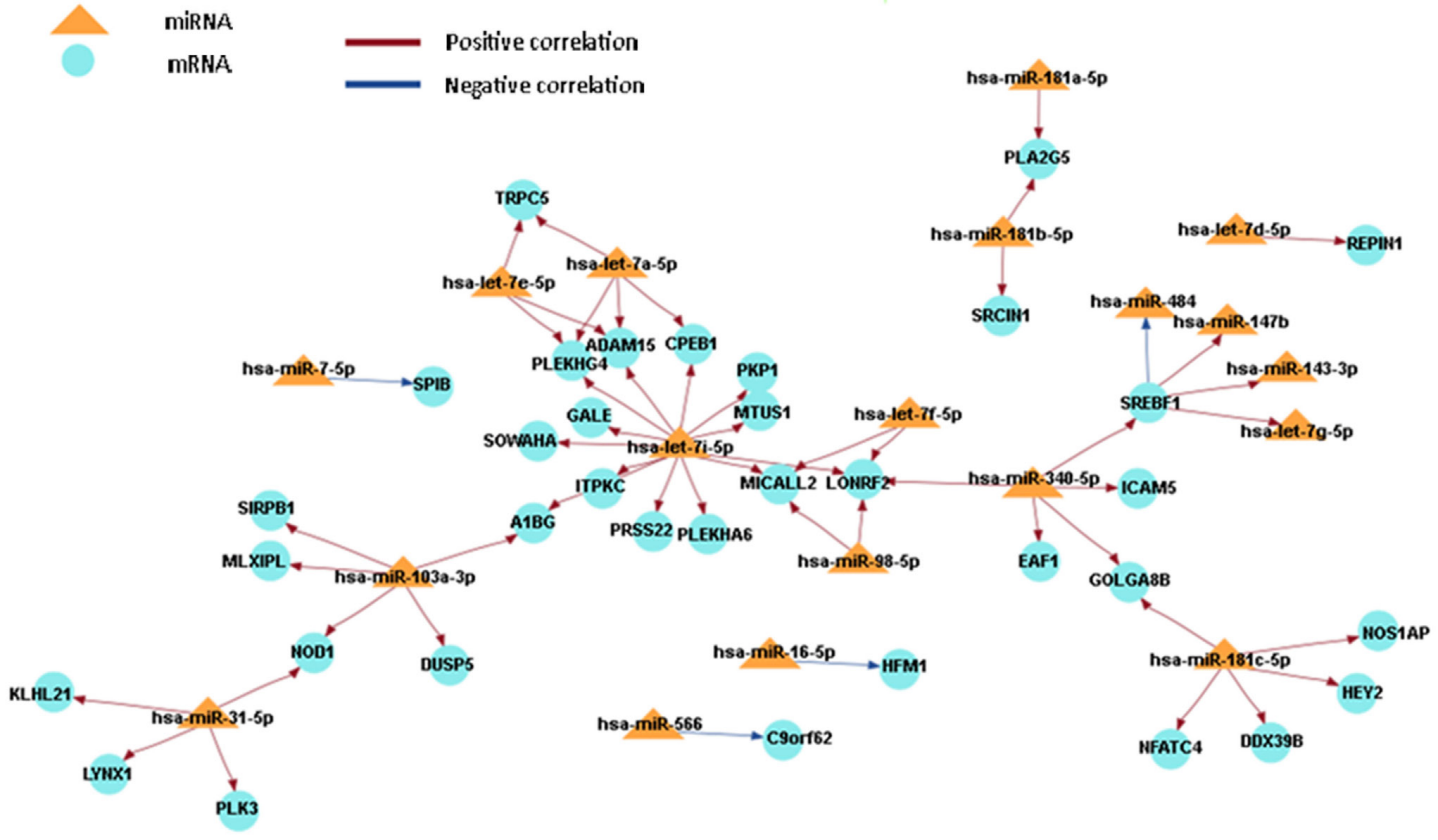
Grand view for 200 main significant microRNA (miRNA)–gene interaction that was generated using miRNA and gene that have significant expression in papillary thyroid carcinoma LNM-P versus their adjacent normal group.

**Figure 7:**
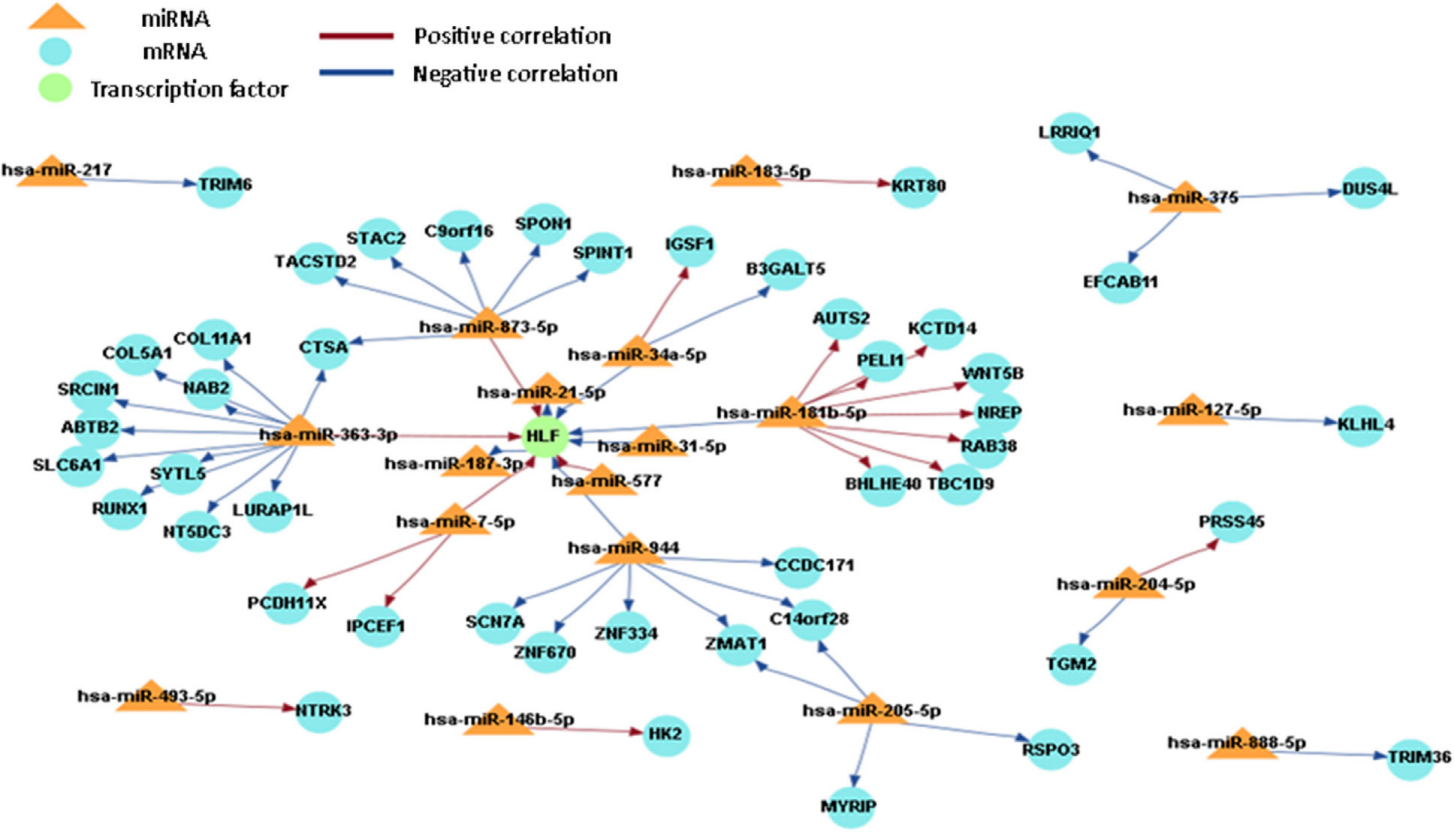
Grand view for 200 main significant microRNA (miRNA)–gene interaction that was generated using miRNA and gene that have significant expression in papillary thyroid carcinoma LNN versus their adjacent normal group.

In order to obtain a comprehensive understanding of regulatory molecules in PTC LNM-P and PTC LNN, integrated analysis of miRNA-gene target was performed using MAGIA2 (23). In PTC LNM-P versus adjacent normal, integrated analysis revealed 47 miRNAs in 295 significant interactions while PTC LNN demonstrated 19 miRNAs in 70 significant interactions. Figures 6 and 7 illustrated 200 top significant miRNA–gene interactions in PTC LNM-P and PTC LNN, respectively. Interestingly, interactions that involved hsa-let-7i-5p and hsa-let-7f-5p only presented in PTC LNM-P.

### Validation of 3p/5p miRNA Expression Ratio

**Figure 8:**
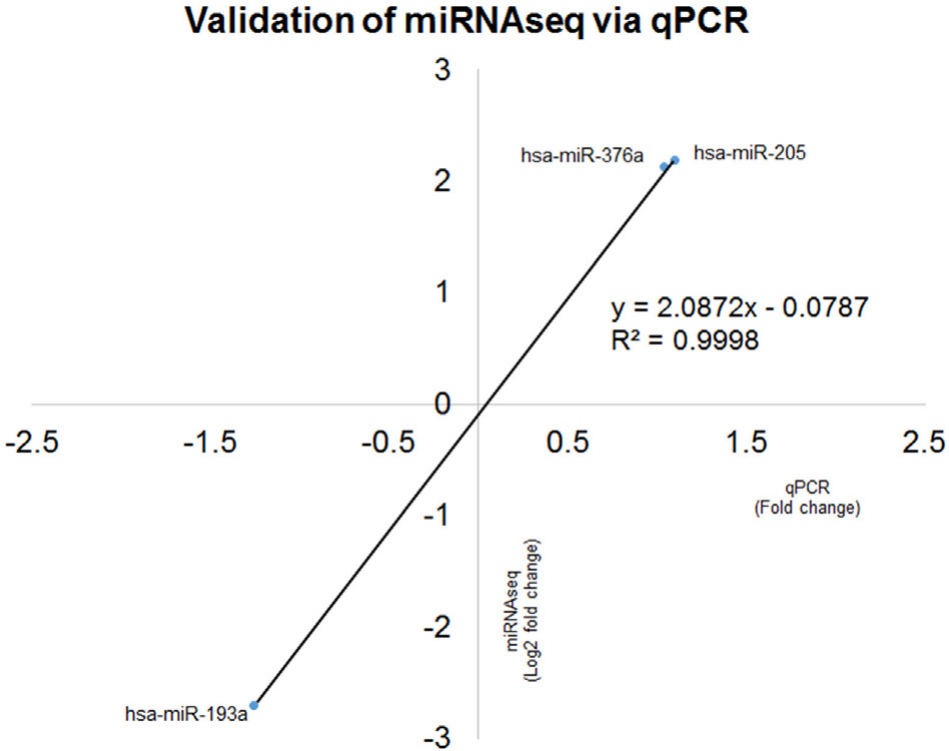
Correlation coefficient analyses for comparison between microRNA (miRNA) sequencing (miRNAseq) and qPCR validation of hsa-miR-193a-3p/5p, hsa-miR-376a-3p/5p and hsa-miR-205-3p/5p. The two technologies produced concordance result with *R*^2^ = 0.9998, indicating the positive correlation of fold change of six selected miRNAs qPCR and miRNAseq.

Expression ratio of selected miRNAs were validated in 21 patients and all were in concordance with miRNA sequencing (*R*^2^ = 0.9998) (Figure 8).

## Discussion

We have identified 138 significant DE miRNAs in PTC LNM-P compared to normal adjacent thyroid tissues; 66 miRNAs were upregulated while 72 miRNAs were downregulated. On the other hand, comparison between PTC LNN and normal adjacent thyroid revealed 43 significant DE miRNAs (31 upregulated and 12 downregulated). A few DE miRNAs from our findings such as hsa-miR-146b, hsa-miR-222, hsa-miR-221, hsa-miR-204, and hsa-miR-7 were commonly dysregulated in both comparisons (PTC LNM-P versus normal adjacent thyroid and PTC LNN versus normal adjacent thyroid) and have consistent expression levels with several other studies (30–33). Hsa-miR-146-5p and 3p in particular are the top upregulated miRNA in PTC compared to normal thyroid and were also highly expressed in our PTC LNM-P and PTC LNN versus respective normal thyroid, suggesting their roles in thyroid carcinogenesis in general. Yet, these miRNAs expression were not significant when we compared PTC LNM-P to PTC LNN. Even though there were no DE miRNAs in PTC LNM-P versus PTC LNN group, we did identified 733 upregulated and 843 downregulated genes. Tables S3–S5 in Supplementary Material summarized the list of significant DE genes. Pathway enrichment analyses of PTC LNM-P revealed alteration in mitogen-activated protein kinase (MAPK) signaling pathway (Figure 3), which could be due to presence of BRAF V600E mutation. On the contrary, this pathway was not enriched in PTC LNN analysis (Figure 4), which does not have BRAF V600E mutation. Our finding is concordant with Wang et al. (34) but is in disagreement with several others (35, 36). On closer inspection, the upregulation was only significant with a larger sample size with a modest fold change. Through functional assays, hsa-miR-146b-5p was shown to promote migration, invasion, and induced epithelial-to-mesenchymal (EMT) by downregulating ZNRF3 and modulating Wnt/β-catenin signaling. However, the function of the passenger strand of miR-146b, hsa-miR-146b-3p, is yet to be elucidated.

Several studies have shown that the 3p/5p miRNA arm selection were variable among human cancers (36, 37). To date, the expression ratio between 3p and 5p miRNA is not well-characterized and there has been only a few studies that investigated the arm selection preference in relation to cancer. Even though 5p and 3p miRNA were derived from the same pre-miRNA, both arms might have different expression in the same tissues where one arm is highly expressed in tumor tissues than normal tissues, or vice versa (36). Our study did not detect any significant DE miRNAs in PTC LNM-P as compared to PTC LNN. However, when we examined 3p and 5p miRNA expression ratio in PTC LNM-P versus PTC LNN, 96 miRNAs have significant expression ratio changes. Among the miRNAs that have significant expression ratios in this study include hsa-miR-205-3p/5p, hsa-miR-193a-3p/5p, and hsa-miR-367a-3p/5p. For example, hsa-miR-205-5p was more highly expressed than hsa-miR-205-3p in both PTC LNM-P and PTC LNN; however, the 3p/5p expression ratio increased from −9.14 in PTC LNN to −6.95 in PTC LNM-P, which is a 2.18 log-fold increment. This suggest that hsa-miR-205-5p was preferentially downregulated in PTC LNM-P while hsa-miR-205-3p expression remained the same. This preference during miRNA biogenesis process could potentially play a role in LNM. To the best of our knowledge, there is only one study previously conducted on 3p and 5p miRNA expression ratio preference in PTC and unfortunately, is inconsistent with our findings (13). Saiselet et al. (13) performed small RNA deep sequencing of only three PTC and compared the miRNA profiles with the matching normal tissues and metastatic lymph node from the same patients. The authors then suggested the lack of involvement of 3p/5p miRNA arm selection in PTC tumorigenesis and LNM progression (13). On the other hands, our analyses involved PTC LNM-P compared to PTC LNN; which were from different patient groups. The inconsistency with study by Saiselet and colleagues (13) could be attributed by different experimental design and this aspect of looking at miRNAs from a different angle certainly warrant further investigations.

Hsa-miR-205 is a regulatory miRNA where its dysregulation can lead to cancer formation, EMT, and metastasis (38). Up until now, there is no study conducted on hsa-miR-205-3p/5p expression ratio in PTC or thyroid cancer in general. However, this miRNA plays two different roles, namely as an oncogene in lung cancer, bladder cancer, cervical cancer and head and neck cancer, and as a tumor suppressor in breast cancer, colorectal cancer and glioma (39, 40). Through our miRNA profiling, hsa-miR-205 was upregulated in PTC LNM-P and PTC LNN when compared to their adjacent normal tissues, respectively. However, only hsa-miR-205-5p reached statistical significance in PTC LNN versus adjacent normal tissues. Our findings were consistent with the study conducted by Nikiforova et al. in 2008 where hsa-miR-205 was upregulated in PTC and anaplastic thyroid cancer as compared to hyperplastic nodule and normal thyroid tissues (41). However, downregulation of hsa-miR-205 was identified to be significant in metastatic PTC as compared to PTC without metastasis. This downregulated expression was associated with upregulation of *VEGF* in thyroid tumor tissue, hence it proved the role of miR-205 as tumor suppressor in a variety of thyroid cancer cell lines (42). These discrepancies and the limited references currently available highlight the needs to further investigate the exact role of miR-205 in PTC and LNM.

In order to identify the role of miRNAs and the pathways involved, pathways enrichment analyses were carried out. Among significant pathways resulted from these analyses were proteoglycans in cancer, ECM–receptor interaction and MAPK signaling pathway. There are 50 genes and 21 miRNAs in PTC LNM-P and 22 genes and 4 miRNAs in PTC LNN that are involved in ECM–receptor interaction pathway. Cellular matrix (ECM) plays an important role in homeostasis development and maintenance as well as tissue and organ architectures. The synthesis and degradation of ECM components such as collagen type I, collagen type IV, and fibronectin can cause modification, proliferation activation, migration, and activated adhesive endothelium cell to ECM and encourage angiogenesis process to occur (43). In a study conducted by Zhang et al. (44), expression of fibronectin-1 was significantly higher in the PTC cell line that was transfected with *CXCR7* and ECM-receptor interaction was one of the enriched pathway resulted. As an important ECM component, *FN1* plays a role in maintaining the survival, proliferation, adhesion, migration, and angiogenesis of epithelial cells (45). In addition, several studies reported that expression of *FN1* was high in PTC and it can act as a potential biomarker in detecting PTC (46, 47). Many other studies have also proven the involvement of ECM in PTC progression (47, 48).

To obtain an understanding of the target mRNA expression that have significant expression, we integrated the miRNA and mRNA sequencing data. When the miRNA acts through the degradation of target mRNA, the miRNA and mRNA target expression profiles are expected to be inversely correlated (49). However, a positive correlation (miRNA highly expressed/mRNA highly expressed or miRNA lowly expressed/mRNA lowly expressed) is formed when the activity of miRNA is part of a series of complex regulatory and gene expression profiles resulting from different levels of regulation (49). There are two miRNAs that are involved in both comparison groups, namely, hsa-miR-7-5p and hsa-miR-181b-5p, hence suggesting that both miRNAs are involved in the pathogenesis of PTC in general and are not specific to LNM. Various studies have reported that hsa-miR-7-5p has a low expression level in PTC samples (13, 31, 50). Hsa-miR-7 plays a role in regulating cell growth, migration, and invasion by targeting *PAK1* in thyroid cancer (51). The low expression level of hsa-miR-181b can cause cell growth inhibition and apoptosis by targeting *CYLD*, and this can be harnessed as a potential therapeutic target for the treatment of patients with PTC (52).

Let-7 (lethal-7) is an important gene involved in development in *C. elegans*, as well as being one of the first miRNA that was discovered (53). Let 7i-5p and let-7f-5p formed a unique miRNA-mRNA network that was only detected in PTC LNM-P versus normal adjacent tissues when the miRNA and mRNA sequencing data were integrated. However, the expression profiling in this study was inconsistent with one previous study (54). The expression of let-7f was not significant in cancer tissues with *BRAF* V600E. However, let-7f was highly expressed in tumor tissues with *BRAF*-wildtype (54). In a study involving breast cancer by Liu et al. (55), let-7f regulated the expression of β2-AR in breast cancer cells. In human breast cancer cell lines (MCF-7, SKBR3, and BT474), let-7f caused an increasing expression of β2-AR and was also shown to be associated with LNM (55). In addition, let-7i was downregulated in PTC with LNM-P compared to PTC LNN (56), and it is upregulated in primary PTC when compared with benign thyroid nodules and normal tissues (39). However, there were no significant changes in the expression when the results were validated using qPCR technique (22).

The T1799A nucleotide transversion in BRAF gene is an oncogenic mutation that is widely reported in PTC, and it occurs in an average of 45% of the cases (57). This mutation causes the change from glutamic acid to valine at codon 600 BRAF protein, resulting in the *BRAF* V600E variant. *BRAF* V600E constitutively increases the activity of serine/threonine protein and activates MAPK signaling pathways in human cancers (57). PTC patients with *BRAF* V600E are often linked with the aggressive clinicopathological characteristics such as LNM (58, 59). In this study, 11 out of 12 PTC LNM-P patients showed the presence of *BRAF* V600E, while it was detected in only two of the nine patients in the PTC LNN samples. From our study, we postulated that the *BRAF* V600E mutation is associated with LNM because almost all (11/12) of PTC LNM-P patients have this mutation. The study conducted by The Cancer Genome Atlas regarding the classification of PTC showed that miRNA expression is associated with the PTC phenotypes and plays an important role in the prognosis of PTC (30). Although definitive studies are still limited, miRNA clearly has the potential as an indicator of prognosis in PTC patients (60). In another study conducted by Cahill et al., which compared a PTC cell line with *BRAF* mutation and a normal thyroid cell line, the analysis revealed 15 highly expressed and 23 downregulated miRNAs (61). In addition, human thyroid cancers that are *BRAF* mutated or have other mutations have different miRNA expression profiles (41). Although the results of our study are consistent with most of other studies (58, 62), there are also other studies, which indicate that *BRAF* V600E mutation do not contribute to the aggressive clinicopathological characteristics in PTC patients (13, 63). These discrepancies could be due to the unique variation in each PTC patient.

## Conclusion

To the best of our knowledge, this is the first study that involved the integrated analysis of miRNA and gene expression profiles from thyroid cancer and the corresponding normal adjacent thyroid tissues in the same patients and associate the expression signatures with LNM and *BRAF* V600E status. PTC LNM-P exhibited a higher number of dysregulated miRNAs and affected pathways compared to PTC LNN. Even though there was no significant DE miRNAs in PTC LNM-P versus PTC LNN, there were significant changes in the 3p and 5p expression ratios. This study proposed the miRNA-gene expression patterns and miRNA 3p/5p ratios that may become potentially useful to identify PTC patients with LNM. Further investigations, such as *in vitro* functional studies are warranted to fully understand the role of these miRNAs in LNM in this cancer.

## Ethics Statement

This study carried out in accordance with the recommendations of UKM Research Ethics Committee (UKMREC; UKM 1.5.3.5/244/UMBI-2015-002). Written informed consent was obtained from all the study participants.

## Author Contributions

AY and N-SM involved in the specimen collections, libraries preparations and sequencings, data analyses, acquisition of data, and drafting the manuscript. RM and SS are thyroid surgeons involved in specimen retrieval. IR assessed tumor percentage of the tissues. SS performed the *BRAF* V600E genotyping. RJ provides critical review on the manuscript. All authors read and approved the final manuscript.

## Conflict of Interest Statement

The authors declare that the research was conducted in the absence of any commercial or financial relationships that could be construed as a potential conflict of interest.
